# An online database for einkorn wheat to aid in gene discovery and functional genomics studies

**DOI:** 10.1093/database/baad079

**Published:** 2023-11-16

**Authors:** Parva Kumar Sharma, Hanin Ibrahim Ahmed, Matthias Heuberger, Dal-Hoe Koo, Jesus Quiroz-Chavez, Laxman Adhikari, John Raupp, Stéphane Cauet, Nathalie Rodde, Charlotte Cravero, Caroline Callot, Inderjit Singh Yadav, Nagarajan Kathiresan, Naveenkumar Athiyannan, Ricardo H Ramirez-Gonzalez, Cristobal Uauy, Thomas Wicker, Michael Abrouk, Yong Q Gu, Jesse Poland, Simon G Krattinger, Gerard R Lazo, Vijay K Tiwari

**Affiliations:** Department of Plant Science and Landscape Architecture, University of Maryland, Fieldhouse Dr. College Park, MD 20742, USA; Plant Science Program, Biological and Environmental Science and Engineering Division, King Abdullah University of Science and Technology (KAUST), 4700 KAUST, Thuwal 23955-6900, Saudi Arabia; Center for Desert Agriculture, KAUST, 4700 KAUST, Thuwal, Kingdom of Saudi Arabia 23955-6900, Saudi Arabia; Department of Plant and Microbial Biology, University of Zurich, 107, Zurich, Zollikerstrasse CH-8008, Switzerland; Wheat Genetics Resource Center and Department of Plant Pathology, Kansas State University, 4024 Throckmorton, 1712 Claflin Road, Manhattan, KS 66506, USA; John Innes Centre John Innes Centre, Norwich Research Park, Norwich NR4 7UH, UK; Plant Science Program, Biological and Environmental Science and Engineering Division, King Abdullah University of Science and Technology (KAUST), 4700 KAUST, Thuwal 23955-6900, Saudi Arabia; Center for Desert Agriculture, KAUST, 4700 KAUST, Thuwal, Kingdom of Saudi Arabia 23955-6900, Saudi Arabia; Wheat Genetics Resource Center and Department of Plant Pathology, Kansas State University, 4024 Throckmorton, 1712 Claflin Road, Manhattan, KS 66506, USA; INRAE, CNRGV French Plant Genomic Resource Center, 24 Chemin de Borde Rouge, Castanet Tolosan F-31320, France; INRAE, CNRGV French Plant Genomic Resource Center, 24 Chemin de Borde Rouge, Castanet Tolosan F-31320, France; INRAE, CNRGV French Plant Genomic Resource Center, 24 Chemin de Borde Rouge, Castanet Tolosan F-31320, France; INRAE, CNRGV French Plant Genomic Resource Center, 24 Chemin de Borde Rouge, Castanet Tolosan F-31320, France; Department of Plant Science and Landscape Architecture, University of Maryland, Fieldhouse Dr. College Park, MD 20742, USA; Supercomputing Core Lab, King Abdullah University of Science and Technology (KAUST), 4700 KAUST, Thuwal 23955-6900, Saudi Arabia; Plant Science Program, Biological and Environmental Science and Engineering Division, King Abdullah University of Science and Technology (KAUST), 4700 KAUST, Thuwal 23955-6900, Saudi Arabia; Center for Desert Agriculture, KAUST, 4700 KAUST, Thuwal, Kingdom of Saudi Arabia 23955-6900, Saudi Arabia; John Innes Centre John Innes Centre, Norwich Research Park, Norwich NR4 7UH, UK; John Innes Centre John Innes Centre, Norwich Research Park, Norwich NR4 7UH, UK; Department of Plant and Microbial Biology, University of Zurich, 107, Zurich, Zollikerstrasse CH-8008, Switzerland; Plant Science Program, Biological and Environmental Science and Engineering Division, King Abdullah University of Science and Technology (KAUST), 4700 KAUST, Thuwal 23955-6900, Saudi Arabia; Center for Desert Agriculture, KAUST, 4700 KAUST, Thuwal, Kingdom of Saudi Arabia 23955-6900, Saudi Arabia; United States Department of Agriculture—Agricultural Research Service, Western Regional Research Center, Crop Improvement and Genetics Research Unit, 800 Buchanan St., Albany, CA 94710, USA; Plant Science Program, Biological and Environmental Science and Engineering Division, King Abdullah University of Science and Technology (KAUST), 4700 KAUST, Thuwal 23955-6900, Saudi Arabia; Center for Desert Agriculture, KAUST, 4700 KAUST, Thuwal, Kingdom of Saudi Arabia 23955-6900, Saudi Arabia; Plant Science Program, Biological and Environmental Science and Engineering Division, King Abdullah University of Science and Technology (KAUST), 4700 KAUST, Thuwal 23955-6900, Saudi Arabia; Center for Desert Agriculture, KAUST, 4700 KAUST, Thuwal, Kingdom of Saudi Arabia 23955-6900, Saudi Arabia; United States Department of Agriculture—Agricultural Research Service, Western Regional Research Center, Crop Improvement and Genetics Research Unit, 800 Buchanan St., Albany, CA 94710, USA; Department of Plant Science and Landscape Architecture, University of Maryland, Fieldhouse Dr. College Park, MD 20742, USA

## Abstract

Diploid A-genome wheat (einkorn wheat) presents a nutrition-rich option as an ancient grain crop and a resource for the improvement of bread wheat against abiotic and biotic stresses. Realizing the importance of this wheat species, reference-level assemblies of two einkorn wheat accessions were generated (wild and domesticated). This work reports an einkorn genome database that provides an interface to the cereals research community to perform comparative genomics, applied genetics and breeding research. It features queries for annotated genes, the use of a recent genome browser release, and the ability to search for sequence alignments using a modern BLAST interface. Other features include a comparison of reference einkorn assemblies with other wheat cultivars through genomic synteny visualization and an alignment visualization tool for BLAST results. Altogether, this resource will help wheat research and breeding.

**Database URL**  https://wheat.pw.usda.gov/GG3/pangenome

## Introduction

Bread wheat (*Triticum aestivum*) is an important staple and the most widely grown food crop in the world. Bread wheat has a hexaploid genome with three subgenomes (ABD) (1). The polyploid journey for hexaploid bread wheat started with a polyploidization event between *Triticum urartu* (A genome) and an *Aegilops speltoides*-related species (B genome) that resulted in tetraploid wild *T. turgidum* ssp. *dicoccoides* (wild emmer, AB genome). The second polyploidization event occurred between tetraploid *T. turgidum* ssp. *dicoccum* and *Ae. tauschii* species (D genome) and resulted in hexaploid *T. aestivum* (2 *n* = 6*x* = 42; ABD) (2, 3).

A recent pangenomic study (4) suggested that modern bread wheat cultivars are genetically as diverse as old landraces. However, to address challenges from human population increase and changing climatic conditions, wheat breeders need to find new sources of genes for various traits including high yield and resistance to biotic and abiotic stresses (5–7). In this context, wild relatives of wheat are a rich source of beneficial genes and are critical for improving wheat cultivars using sustainable approaches (8–11). The diploid ‘A’ genome progenitor gene pool of wheat has two closely related species *T. monococcum* [(*T. monococcum* L. subsp. *monococcum* (domesticated), *T. monococcum* L. subsp. *aegilopoides* (wild)] and *T. urartu* (wild). As mentioned earlier, these species harbor various agronomically important traits for wheat improvement. Although *T. monococcum* is not the direct donor of the bread wheat A-genome, it shares high homology with the A-genome of present-day cultivated hexaploid and tetraploid wheat and gene transfers are feasible between bread wheat and *T. monococcum*.

Einkorn is considered as a relict crop, being replaced over time by tetraploid and hexaploid landraces and then modern varieties. Its gene pool being different from the bread wheat gene pool presumably contains novel alleles. Einkorn wheat has been used as a source of genetic variation for wheat breeding (12). Despite the importance of einkorn wheat in wheat breeding, there is a lack of resources for finding highly reliable genetic and genomic datasets on einkorn wheat as well as its genome organization.

A recent work completed by Ahmed *et al*. (13) presents an exciting resource for the cereals genetics and breeding community. Presented here is an online database to accompany these data organized in a manner to assist comparative genomics, applied genetics and breeding research. There is a need to supply quality assembly data for the *T. monococcum* A-genome and integrate it with other available wheat reference genomes and datasets. Reported here is information about a database resource created for the einkorn reference genomes, the work presented by Ahmed *et al*. 2023 [raw data available from NCBI SRA resource as PRJEB52766 (TA10622) and PRJEB52767 (TA299)]. Annotations and other analysis are available from the Ahmed *et al.* (2023) Supplementary Material.

Here, we have integrated various tools and genomic resources allowing us to compare the einkorn genomes with reference assemblies of 27 other wheat accessions and their respective annotations from wild, related and cultivated germplasm at a common platform. The goal of this research was to provide easy visualization and analysis of these genomic resources to answer critical biological questions using various tools that have been integrated into the database. This database is currently hosted alongside GrainGenes (https://wheat.pw.usda.gov/GG3/pangenome) (14) resources where it can be useful for the wider small grain cereal genetics, genomics and breeding community.

## Materials and Methods

### Data Source

**Figure 1.**
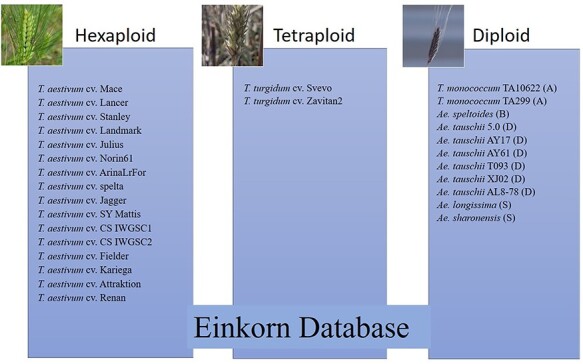
List of hexaploid, tetraploid and diploid wheat species in the pan-genome BLAST database and synteny comparison. Among the total 29 varieties, 16 are hexaploid, two are tetraploid and 11 are diploid (two with A genome, one with B genome, six with D genome and two with S genome).

The data used and presented in this database is associated with a recently published manuscript on *T. monococcum* (13). Along with einkorn datasets (two genomes), this database uses publicly available data for 27 wheat lines (diploid, tetraploid, and hexaploid wheat species) and the description of these lines/accessions are presented in Supplementary Table 1. Out of these 29 varieties, 16 are hexaploid (15–19), two are tetraploid (20, 21) and the remaining 11 are diploid (22–24) (Figure 1). For the genome browser, *T. monococcum* genomes from two accessions (TA10622 and TA299) and their associated annotations (gene structure, function and transposable elements) were indexed and uploaded into the queryable search tool to generate links to the genome browser and a search-based BLAST database for all 29 wheat lines included.

### Database implementation

This database has been designed using the standard LAMP protocol, i.e. Linux (RHEL8), Apache (2.4.37), MariaDB (10.5.16) and PHP (8.0.13) (Hypertext Preprocessor). This is a relational database based on a ‘three-tier architecture’ having a client-, middle- and database tier, that catalogs the information related to *T. monococcum*.

The process starts when a user submits a query through the ‘client tier’, where interactive web pages have been developed using HTML and Bootstrap5. Bootstrap5 is the latest version in its legacy and provides multi-device usability and responsiveness. For the user queries, fetching, and execution, scripting in PHP has been done in the ‘middle tier’ along with HTML, PHP and Javascript. This PHP acts as a bridge between the user and the database. Any query submitted by the user is first processed by the PHP scripts into SQL queries and then forwarded to the database search engine.

The ‘database tier’ holds all the data like gene ids, chromosome numbers, positions, gene sequences and other likewise data in the form of relational tables in the MariaDB database. The query coming from the middle tier is then searched in the database table and the results are given back to the middle tier that processes the results to be displayed to the user. We have used the latest tools and packages for data visualization and analysis. Still, the majority of the online available resources use older versions of tools that have less functionality and features.

### Integrated tools and Web Interface

**Figure 2.**
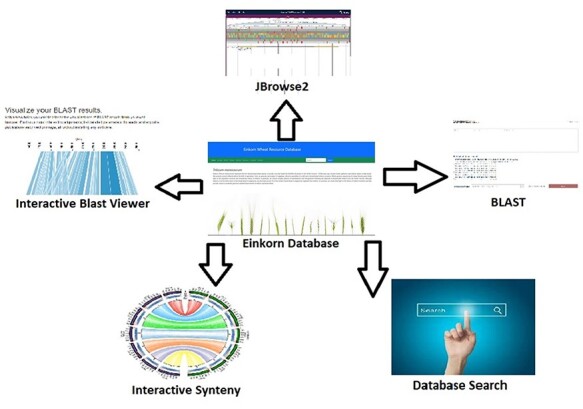
Interactive tools such as JBrowse2, BLAST database search, database search and synteny have been incorporated within the Einkorn database.

Analysis, interpretation, and presentation of raw data in a user-friendly manner always help in getting useful outcomes. Software/tools help in processing and analyzing data and hence result in meaningful inferences. In the present database, we have integrated several tools which help in data analysis (Figure 2). JBrowse is a genome browser tool used to navigate through the genome and its annotations. It’s version 2 (JBrowse2) which is a recent release (25) and harbors many new functionalities like circular view, linear comparative view, linear synteny view, and structural variant Inspector view (SvInspectorView). Another important tool is SequenceServer2 which is a recently updated and released BLAST engine. It takes the FASTA-formatted sequences as queries and aligns them to the selected BLAST databases. Its output is quite interactive and informative (26).

We have also used a couple of online tools to visualize the data. Kablammo (27) is a BLAST result output viewer. Kablammo accepts BLAST result output files in XML format and will illustrate exactly which portions of the query sequence mapped to which portions of the subject sequence. The other online tool that is used in the database is AccuSyn (28). AccuSyn is interactive software that shows circular syntenic plots of chromosomes and draws links between similar blocks of genes using Simulated Annealing to minimize link crossings. This helps researchers with insights into the evolutionary history of species or functional relationships between genes. This tool requires two input files *viz.* an annotation file (.gff) and an alignment file generated from McScanX (29).

**Figure 3.**
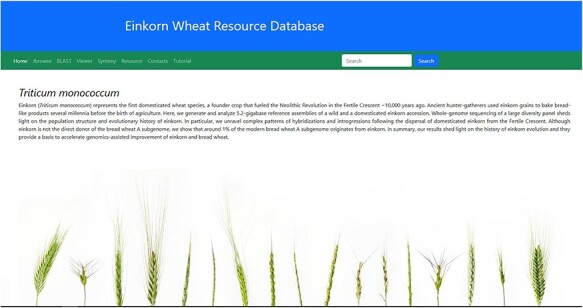
Home page of the einkorn wheat resource database.

An interactive webpage has been designed for this database. All the above-mentioned tools have been put together in respective tabs on the homepage of the database (Figure 3).

## Results

This database has been developed to analyze and visualize the *T. monococcum* reference quality assemblies. For this purpose, various tools and search elements have been integrated with the database. The following section will describe all the integrated tools along with their usability.

### JBrowse2

**Figure 4.**
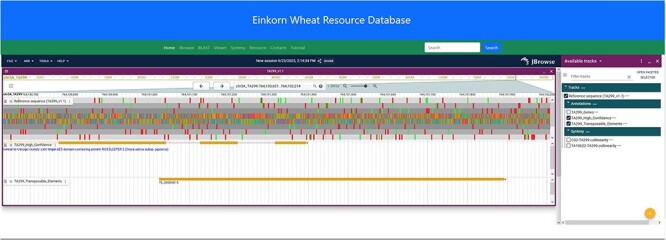
JBrowse2 Linear genome viewer page showing *T. monococcum* TA299 assembly, a gene structure, and a transposable element. Users can scroll through the genome and can see available tracks from the panel on the right.

JBrowse2 is the latest release of the JBrowse Genome Browser (25). Previously released Jbrowse1 (30, 31) had some limitations like only one genome would be displayed and needed more support for new software libraries. Here, we have used the linear genome viewer of JBrowse2 to show the genome assemblies of *T. monococcum* TA299 and *T. monococcum* TA10622 (Figure 4). Both assemblies can be accessed by individual chromosomes. Gene model annotations were linked to each of the assemblies which include gene structure and putative function for both high-confidence and low-confidence genes models. However, a separate annotation file has been provided for only high-confidence genes for those who are interested in high-confidence genes. The annotated transposable elements (TEs) were also associated with each of the genomes.

**Figure 5.**
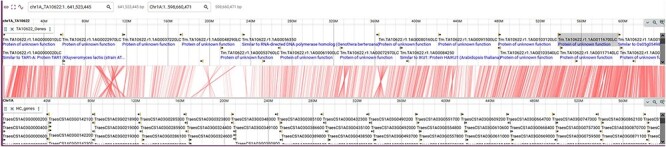
JBrowse2 linear synteny viewer showing synteny between *T. monococcum* TA10622 and *T. aestivum* CS-IWGSC ref2.1. See tutorial on online webpage (https://avena.pw.usda.gov/genomes/mono/pan_help.php) on how to generate this view in JBrowse2.

Another new feature in Jbrowse2 is the linear synteny viewer. This is quite an informative feature that shows synteny between two genomes. Here we have provided the pairwise synteny between *T. monococcum* TA10622, *T. monococcum* TA299 and *T. aestivum* CS-IWGSC ref2.1. Figure 5 shows an example of such synteny between chromosome 1A of *T. monococcum* TA10622 and *T. aestivum* CS-IWGSC ref2.1.

### SequenceServer2

SequenceServer2 is a recent web-based service application for the display of nucleic acid- and protein-based sequence alignments (26). It makes use of the widely accepted BLAST application (32, 33) and has been upgraded to accept the optimized algorithms in use with the present version used here (2.13.0+). This provides output in a table that can be downloaded. Other download options like XML and alignment format are also available.

**Figure 6.**
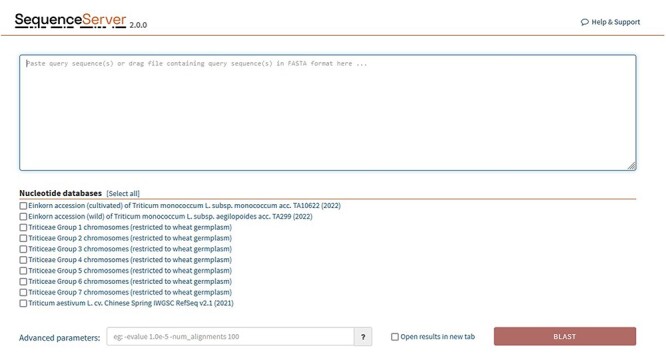
SequenceServer2 page for BLAST search. Specific BLAST databases have been provided for searching specific chromosome groups of all 29 wheat varieties studied.

BLAST databases are provided for the following genomes: (Figure 6).

*Triticum monococcum* L. subsp. *aegilopoides* acc. TA299 (wild einkorn) (2022).

*Triticum monococcum* L. subsp. *monococcum* acc. TA10622 (domesticated einkorn) (2022).

*Triticum aestivum* L. cv. Chinese Spring IWGSC RefSeq v2.1 (2021).

Triticeae groups 1–7 chromosomes are listed individually and are restricted at present to 29 wheat germplasm (Supplementary Table S1).

**Figure 7.**
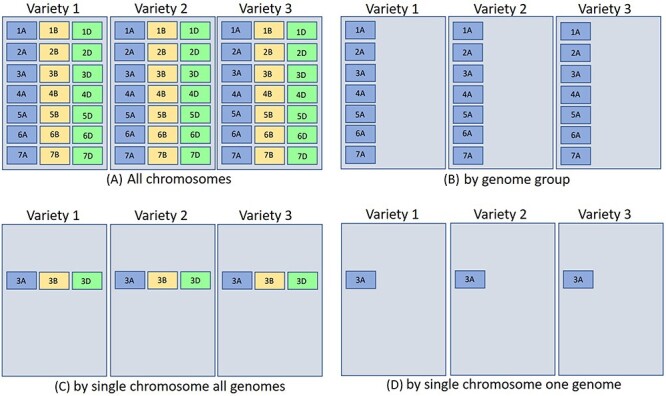
The above represents three different hexaploid wheat cultivars BLAST database sets searched by four different parameters [only the (a) quadrant contains all chromosomes for hexaploid wheat]. The other quadrants are partitioned by (b) genome group (1–7A genome), (c) single chromosome all genomes (3ABD), and (d) single chromosome single genome (3A). Database indexing partitioned in such a fashion for (b–d) takes less time and resources (see text for more explanation). For wheat, each chromosome block would represent about 700 Mb.

The concept of groups is interesting and unique; here, we have indexed our BLAST databases in a chromosome-wise fashion (Figure 7), such that a user will be able to search against A, B and D genomes restricted to a particular chromosome number. For example, checking the Group 1 option, a search can be performed against chromosome 1 of all the A, B and D genomes of 29 wheat varieties and so on. Another important aspect of the ‘grouping’ concept is the use of this method in pan-genome analysis to access diversity across many individuals in a species rather than a single reference genome. For constructing a pan-genome, all the available varieties (reference quality assemblies) of a species are considered and hence making a pan-genome quite large. Searching/querying this large dataset can be time and resource consuming. Using the ‘Groups’ only selection, a desired subset of the pan-genome BLAST database can be queried. This will save time and resources.

The use of the web interface depends on many factors, such as the web browser used, internet connections and possibly other computer configurations. The interface provides selecting more than one database for searches, but here we recommend selecting only one-database-at-a-time. If more than one database will be searched, it is best to select the checkbox ‘Open results in a new tab’ located at the bottom of the page near the BLAST submit button; this will allow the original query sequence to be used for multiple database queries. Likewise, to get the best use of the databases provided it is also advisable to only include one-sequence-at-a-time. If one sequence and one database are selected, each BLAST submission will open a new tab on the browser.

As an example, using a sample sequence (TraesCS5A01G000200, a trypsin family protein-encoding gene) a search will yield strong matches in the *T. monococcum* accessions, with strong matches on the 5A chromosome at the telomeric 5ʹ-end and weaker matches on the 2A chromosome also at the 5ʹ-end, but not so telomeric. A further search of the same sequence against the Chinese Spring (CS) reference will also confirm observations seen with the A genome, and also note similar alignments in the B and D genomes; in this case, alignments were stronger in 5A, with identity ranking 5A > 5B > 5D (the test sequence was derived from CS 5A); and the weaker alignments in chromosome 2 ranking 2B > 2A > 2D.

### BLAST results viewer (Kablammo)

**Figure 8.**
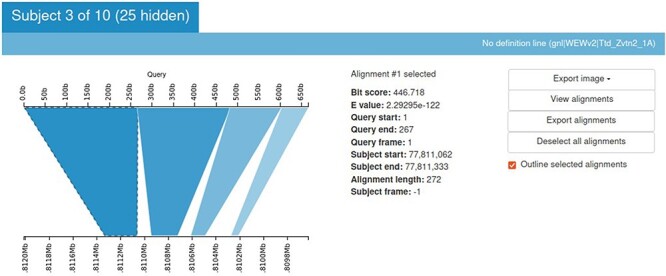
Kablammo display for the BLAST results of a sample query sequence. Various alignment statistics are being shown along with the option to view/download images and alignments. Please see the tutorial on the einkorn resource database webpage on how to generate this output (https://avena.pw.usda.gov/genomes/mono/pan_help.php).

Sequence Server provides the BLAST output in an output table. But it is always more informative to look at an image. Kablammo is a tool that facilitates the viewing of the BLAST results interactively (27). Kablammo takes an XML file as an input (which can be downloaded from SequenceServer output) and provides an interactive figure for each subject matched. The query as well as subject sequences can be zoomed-out or zoomed-in for better results viewing experience. This is helpful to detect INDELs or duplications. Other features like image export, alignment viewer and exporter are also available (Figure 8).

### Synteny viewer

**Figure 9.**
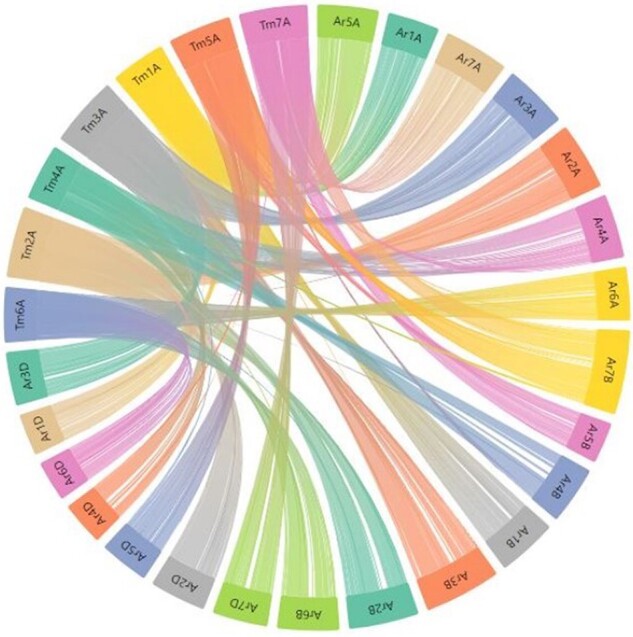
Part of the webpage shows a synteny between the *T. monococcum* TA10622 and *T. aestivum* ArinaLrFor. The figure is available on the synteny tab of einkorn wheat resource database web page by selecting the respective genome. This figure can also be generated by uploading the two precomputed files (gff and collinearity) provided on the synteny tab of einkorn resource database web page on the AccuSyn server.

In the present relational database, we have provided pre-computed synteny results for 29 wheat varieties with the domesticated *T. monococcum* accession TA10622. These results can be viewed by selecting the relevant variety from the dropdown menu on the left side of the synteny tab on the webpage. Users can have an overview of the synteny between the TA10622 and other wheat varieties. An example of synteny between *T. monococcum* TA10622 and *T. aestivum* cultivar ArinaLrFor is shown in Figure 9. However, many researchers are particularly interested in one or two chromosomes and want to look into the synteny of only those chromosomes. We have therefore provided the input files (GFF and collinearity files) to be uploaded into AccuSyn, an online interactive software to view the synteny (28). Users can download these zipped files for different wheat varieties from the dropdown list provided on the right side of the synteny tab. Then they need to extract and upload the two files to the Accusyn website, for which a link has been provided on the same tab. AccuSyn displays synteny between genomes by circularly displaying chromosomes (Figure 9). A user can select one or more chromosomes as required. An interactive figure on the right displays the blocks that were matched between the chromosomes. It should be noted that the same input files can be used as input in SynVisio (https://synvisio.github.io) which can display synteny in a linear fashion which is useful for viewing a few chromosomes at a time.

**Figure 10.**
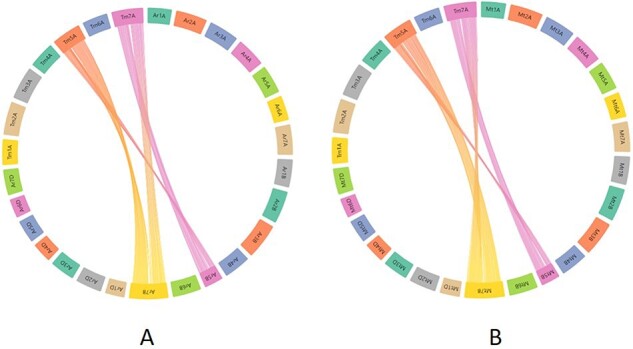
Synteny visualization of *T. monococcum* with Arina*LrFor* (A) and SY Mattis (B). It shows the presence of a translocation between chromosomes 5B and 7B of Arina*LrFor* (A) and SY Mattis (B). The figure was generated by uploading the two precomputed files (gff and collinearity) provided on the synteny tab of einkorn resource database web page and viewed on the AccuSyn server by selecting the respective chromosomes.

To assess the practical applicability of this synteny tool, we took an example from the recent bread wheat pan-genome paper (4). In this paper, the authors studied the translocation between chromosomes 5B and 7B in bread wheat lines Arina*LrFor* and SY Mattis. As our synteny analysis maps/relates the *T. monococcum* A genome to all the A, B and D genomes of hexaploid wheat, we wanted to look into the aforementioned 5B/7B translocation through our synteny tool. For this, the zipped files provided for Arina*LrFor* and SY Mattis were used for visualization purposes. Figure 10A shows the synteny view of Arina*LrFor* chromosomes 5B and 7B with *T. monococcum* chromosome 5A. This shows the presence of a translocation between Arina*LrFor* chromosome 5B and 7B through our synteny analysis. The same phenomenon was also observed with wheat variety SY Mattis chromosome 5B and 7B.

### Database searching

**Figure 11.**
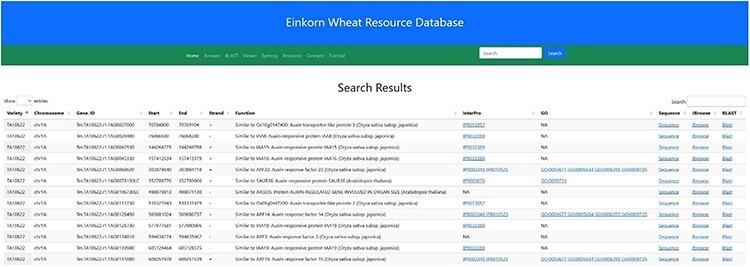
Search result for the search term ‘auxin’. Searching the relational database for any auxin-related genes yielded 338 possible hits. Links to InterPro, Gene Ontology, Gene sequence, JBrowse2 and Blast results are provided in the subsequent columns.

In the navigation bar, a search box has been incorporated by which users can search either by a particular gene ID or any probable candidate gene function keyword. As of now, the gene ID and function searches are restricted to two *T. monococcum* accessions (TA10622 and TA299). As an example, searching for the keyword ‘auxin’ will result in 338 table entries having auxin as a keyword in their function (Figure 11). This table along with the putative function also displays the accession, chromosome number, Gene ID, Gene Ontology (GO), start and end position and the DNA strand.

Links have been provided in the table to view the fasta sequence of that particular gene, to view the gene in JBrowse2, and to view the dashboard summarizing the statistics of BLAST results of that gene with other wheat varieties (from a pan-genomic perspective).

**Figure 12.**
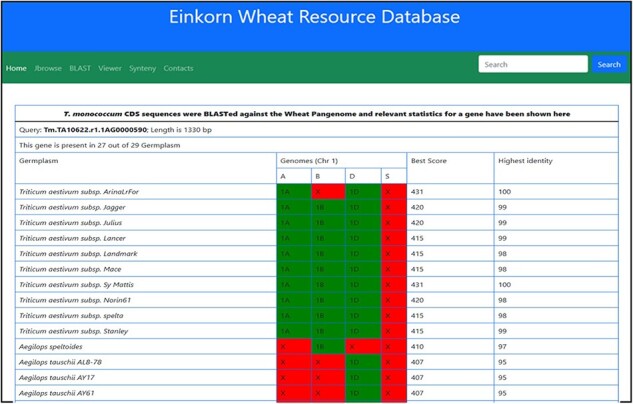
Table showing the BLAST statistics of a gene against the wheat pan-genome collection. This table is accessible from the Blast column of search results table.

This dashboard provides various statistical informations derived from the BLAST of *T. monococcum* genes against the other wheat varieties. The statistics provided here include the length of gene sequence, number of germplasm matched along with the individual genomes, length matched, scores, etc. (Figure 12).

### A case study

*Sr35* is the first gene cloned for stem rust resistance, encoding an intracellular immune receptor of the nucleotide-binding site—leucine-rich repeat (NLR) protein family (34). This gene originates from *T. monococcum* chromosome 3A. The gene sequence (3151 bp) of *Sr35* was BLAST searched against the wheat pangenome using a chromosome group-wise search. Hits were seen in all the chromosome groups except group 4 (Table 1). Significant matches with high identity were found on chromosome 3 in all the 57 genome entities matched (Supplementary Figure S1). However, screening each alignment yielded unique sequences/patterns in different cultivars. This information was particularly useful in marker-assisted selection for stem rust.

**Table 1.** Table showing the BLAST statistics of the *Sr35* gene against the wheat pan-genome collection

Chromosome groups	Genomic hits	Genome groups
Group1	1	B
Group2	21	DS
Group3	57	ABDS
Group4	No matches	—-
Group5	17	BD
Group6	14	SB
Group7	41	BAD

Filtering the blast results with sequence identity >90% and query coverage >95% (from Group 3 hits), removed most of the non-significant hits, including the B sub-genome matches. Significant blast hits on A-genome consisted of genomes, i.e. *T. monococcum* L. subsp. *aegilopoides* (TA299), tetraploid wheat accession Zaviton, and wheat cv. ArinaLrFor, Norin, Mattis, Mace, Jagger, Julius, Landmark, Kariega *and T. spelta*, after removal of D-genome counterparts. For sequence comparison, we extracted 500 bp extended sequences from the respective cultivars. The sequence identity ranges from 91% to 96% between the *Sr*35 and wheat cultivars. Supplementary Figure S4 shows the multiple sequence alignment of the *Sr*35 coding gene with the selected genomes. An insertion in the Fielder and ArinalLrFor genome was observed. The phylogenetic tree showed two distinct groups (Supplementary Figure S5). These two groups differed in terms of SNP and Indel. To demonstrate the similarity at the protein level, we performed gene prediction in the regions, which showed wide differences in the gene structure, with the number of predicted exons ranging from 1 to 4, making these targets as pseudo genes with respect to *Sr*35 reference. *Sr*35 seems to have a unique introgression, with missing or pseudo-gene homologs between the cultivars.

A close inspection in the case of *T. monococcum* TA299 helped to reveal two significant matches (Supplementary Figure 2) on chromosome 3A. Both the two matches were within a 30 kb region at position 770.4 Mb. We then used JBrowse2 to look into the corresponding positions on the assembly with annotations. Both the two regions matched have only a single type of disease-resistant gene, i.e. ‘Disease resistance protein RGA5’ (Supplementary Figure S3). However, it is interesting to note that around this position there are a lot of other insignificant matches that were found which again support the presence of pseudogenes around this position.

The above case study is just one example of how the multi-genome database can be used to compare gene sequences. The possibilities for gene discoveries within the database are plentiful.

## Discussion

We have recently seen a surge in high-quality crop reference genomes. This increase has raised the urgent need to effectively integrate and process the data to be easily used by researchers. In the case of wheat, the high-quality Chinese Spring latest assembly is included (35). Also, the diploid wild donor species like *Ae. speltoides* (36) and *Ae. tauschii* (37) have been sequenced and the browsable sequences are available at the GrainGenes website (https://wheat.pw.usda.gov). Until recently, a high-quality *T. monococcum* assembly was lacking despite its importance in wheat improvement. To address this, Ahmed *et al.* (2023) (13) have recently provided a high-quality *T. monococcum* assembly. Taking this opportunity, we have decided to put together this assembly with other high-quality wheat assemblies at a common place to be utilized by researchers.

*T. monococccum* shares a close homology with the *T. urartu* (‘A’ genome donor). Moreover, *T. monococcum* being a domesticated accession makes this species ideal for breeding programs and variety development. A reference quality genome assembly assists in identifying accurate alleles for agronomically important traits. In the present paper, we have tried to harness the potential of this assembly and related annotations from a pan-genomic perspective. We have used 29 wheat varieties (diploid, tetraploid and hexaploid) in the database which corresponds to 63 genomes (20 A, 19 B, 22 D and 2 S genomes).

This database has several useful features which enhance the users’ experience in analyzing and visualizing genomes. For example, the circular synteny viewer provides a syntenic overview of the various wheat varieties with *T. monococcum*. Synteny is an important analysis as it allows us to look into a bigger perspective of chromosome-scale rearrangements like translocations and large INDELS. Also, on a gene scale, it was able to detect gene duplication and structural variants. With wheat’s polyploid nature and its highly repetitive genome, syntenic analysis and visualization play a crucial role in understanding the many important evolutionary changes including translocations and sequence arrangements. The einkorn genome analysis (13) describes the synteny between two *T. monococcum* accessions (TA299 (wild) and TA10622 (domesticated)) and also with the A sub-genome of bread wheat. However, looking from a pan-genome perspective, it is important to look into other wheat varieties while looking into not only the A sub-genome but also the B and D subgenomes. Another important tool that we have incorporated is JBrowse2 which is a modern upgrade and can display multiple genomes. Along with all the basic functionalities of JBrowse1, JBrowse2 has added the syntenic visualization functionalities for visualizing structural variants in genomes and evolutionary relationships among genes and genomes. Other tools include BLAST search and BLAST result viewer. All of these will assist in querying, searching and visualizing the gene sequences.

The database as presented in this manuscript is work in progress that will be continuously updated and improved. In future, we plan to add genomes from other Triticeae species such as rye, barley and oat. Including the genomes of these members will enhance our understanding in terms of evolutionary and pan-genomic viewpoints. New tools for analysis and visualization will also be incorporated. We plan to update the database regularly, every six months in terms of updated genome versions and high-quality assemblies.

In the einkorn database, the large number of high-quality wheat genomes from multiple varieties, together with highly efficient analysis and visualization tools, will greatly assist the researchers in uncovering relevant questions. Overall, this database is an effort to take the first step towards a comprehensive pan-genomic database.

## Supplementary Material

baad079_Supp

## Data Availability

No new data were generated or analyzed in support of this research. The database is available at https://wheat.pw.usda.gov/GG3/pangenome.

## References

[1] Pont C. , Leroy T., Seidel M. et al. (2019) Tracing the ancestry of modern bread wheats. *Nat. Genet.*, 51, 905–911. 31043760 10.1038/s41588-019-0393-z

[2] Dvorak J. , Luo M.C., Yang Z.L. et al. (1998) The structure of the Aegilops tauschii genepool and the evolution of hexaploid wheat. *Theor. Appl. Genet.*, 97, 657–670.

[3] Luo M.C. , Yang Z.L., You F.M. et al. (2007) The structure of wild and domesticated emmer wheat populations, gene flow between them, and the site of emmer domestication. *Theor. Appl. Genet.*, 114, 947–959. 17318496 10.1007/s00122-006-0474-0

[4] Walkowiak S. , Gao L.L., Monat C. et al. (2020) Multiple wheat genomes reveal global variation in modern breeding. *Nature*, 588, 277–283. 33239791 10.1038/s41586-020-2961-x PMC7759465

[5] Iizumi T. , Furuya J., Shen Z.H. et al. (2017) Responses of crop yield growth to global temperature and socioeconomic changes. *Sci. Rep.*, 7, 7800. 10.1038/s41598-017-08214-4 PMC5552729 28798370

[6] Ray D.K. , Mueller N.D., West P.C. et al. (2013) Yield trends are insufficient to double global crop production by 2050. *PloS One*, 8, e66428. 10.1371/journal.pone.0066428 PMC3686737 23840465

[7] Ray D.K. , Ramankutty N., Mueller N.D. et al. (2012) Recent patterns of crop yield growth and stagnation. *Nat. Commun.*, 3, 1293. 10.1038/ncomms2296 23250423

[8] Dempewolf H. , Baute G., Anderson J. et al. (2017) Past and future use of wild relatives in crop breeding. *Crop Sci.*, 57, 1070–1082.

[9] Kilian B. , Dempewolf H., Guarino L. et al. (2021) Crop Science special issue: Adapting agriculture to climate change: A walk on the wild side. *Crop Sci.*, 61, 32–36.

[10] Placido D.F. , Campbell M.T., Folsom J.J. et al. (2013) Introgression of novel traits from a wild wheat relative improves drought adaptation in wheat. *Plant Physiol.*, 161, 1806–1819. 23426195 10.1104/pp.113.214262 PMC3613457

[11] Zhang H.Y. , Mittal N., Leamy L.J. et al. (2017) Back into the wild-apply untapped genetic diversity of wild relatives for crop improvement. *Evol. Appl.*, 10, 5–24. 28035232 10.1111/eva.12434 PMC5192947

[12] Zohary D. , Hopf M. and Weiss E. (2012) *Cereals. Domestication of Plants in the Old World: The Origin and Spread of Domesticated Plants in South-West Asia, Europe, and the Mediterranean Basin*. 4th edn. Oxford University Press, Oxford, pp. 20–74.

[13] Ahmed H.I. , Heuberger M., Schoen A. et al. (2023) Einkorn genomics sheds light on history of the oldest domesticated wheat. *Nature*, 620, 830–838. 37532937 10.1038/s41586-023-06389-7 PMC10447253

[14] Yao E. , Blake V.C., Cooper L. et al. (2022) GrainGenes: a data-rich repository for small grains genetics and genomics. *Database (Oxford)*, 2022, baac034. 10.1093/database/baac034 PMC9216595 35616118

[15] International Wheat Genome Sequencing, C (2018) Shifting the limits in wheat research and breeding using a fully annotated reference genome. *Science*, 361, eaar7191. 10.1126/science.aar7191 30115783

[16] Sato K. , Abe F., Mascher M. et al. (2021) Chromosome-scale genome assembly of the transformation-amenable common wheat cultivar ‘Fielder’. *DNA Res.*, 28, dsab008. 10.1093/dnares/dsab008 PMC8320877 34254113

[17] Athiyannan N. , Abrouk M., Boshoff W.H.P. et al. (2022) Long-read genome sequencing of bread wheat facilitates disease resistance gene cloning. *Nat. Genet.*, 54, 227–231. 35288708 10.1038/s41588-022-01022-1 PMC8920886

[18] Kale S.M. , Schulthess A.W., Padmarasu S. et al. (2022) A catalogue of resistance gene homologs and a chromosome-scale reference sequence support resistance gene mapping in winter wheat. *Plant Biotechnol. J.*, 20, 1730–1742. 35562859 10.1111/pbi.13843 PMC9398310

[19] Aury J.M. , Engelen S., Istace B. et al. (2022) Long-read and chromosome-scale assembly of the hexaploid wheat genome achieves high resolution for research and breeding. *Gigascience*, 11, giac034. 10.1093/gigascience/giac034 PMC9049114 35482491

[20] Maccaferri M. , Harris N.S., Twardziok S.O. et al. (2019) Durum wheat genome highlights past domestication signatures and future improvement targets. *Nat. Genet.*, 51, 885–895. 30962619 10.1038/s41588-019-0381-3

[21] Zhu T.T. , Wang L., Rodriguez J.C. et al. (2019) Improved Genome Sequence of Wild Emmer Wheat Zavitan with the Aid of Optical Maps. *G3 Genes Genom. Genet.*, 9, 619–624. 10.1534/g3.118.200902 PMC6404602 30622124

[22] Zhou Y. , Bai S.L., Li H. et al. (2021) Introgressing the Aegilops tauschii genome into wheat as a basis for cereal improvement. *Nat. Plants*, 7, 774–786. 34045708 10.1038/s41477-021-00934-w

[23] Luo M.C. , Gu Y.Q., Puiu D. et al. (2017) Genome sequence of the progenitor of the wheat D genome Aegilops tauschii. *Nature*, 551, 498–502. 29143815 10.1038/nature24486 PMC7416625

[24] Yu G. , Matny O., Champouret N. et al. Reference genome-assisted identification of stem rust resistance gene Sr62 encoding a tandem kinase. PREPRINT (Version 1) available at Research Square. https://www.researchsquare.com/article/rs-1198968/v1 (29 December 2021, date last accessed).

[25] Diesh C. , Stevens G.J., Xie P. et al. (2023) JBrowse 2: a modular genome browser with views of synteny and structural variation, *Genome Biol.*, 24, 74. 10.1186/s13059-023-02914-z PMC10108523 37069644

[26] Priyam A. , Woodcroft B.J., Rai V. et al. (2019) Sequenceserver: a modern graphical user interface for Custom BLAST databases. *Mol. Biol. Evol.*, 36, 2922–2924. 31411700 10.1093/molbev/msz185 PMC6878946

[27] Wintersinger J.A. and Wasmuth J.D. (2015) Kablammo: an interactive, web-based BLAST results visualizer. *Bioinformatics*, 31, 1305–1306. 25481007 10.1093/bioinformatics/btu808

[28] Jorge Núñez S. , Eric N., Isobel P. et al. (2020) Using simulated annealing to declutter genome visualizations. In: *Florida Artificial Intelligence Research Society Conference; The Thirty-Third International Flairs Conference*, Florida, May 17-20, 2020, 201–204.

[29] Wang Y.P. , Tang H.B., DeBarry J.D. et al. (2012) MCScanX: a toolkit for detection and evolutionary analysis of gene synteny and collinearity. *Nucleic. Acids Res.*, 40, e49. 10.1093/nar/gkr1293 PMC3326336 22217600

[30] Skinner M.E. , Uzilov A.V., Stein L.D. et al. (2009) JBrowse: a next-generation genome browser. *Genome Res.*, 19, 1630–1638. 19570905 10.1101/gr.094607.109 PMC2752129

[31] Buels R. , Yao E., Diesh C.M. et al. (2016) JBrowse: a dynamic web platform for genome visualization and analysis. *Genome Biol.*, 17, 66. 10.1186/s13059-016-0924-1 PMC4830012 27072794

[32] Altschul S.F. , Gish W., Miller W. et al. (1990) Basic local alignment search tool. *J. Mol. Biol.*, 215, 403–410. 2231712 10.1016/S0022-2836(05)80360-2

[33] Camacho C. , Coulouris G., Avagyan V. et al. (2009) BLAST+: architecture and applications. *BMC Bioinform.*, 10, 421. 10.1186/1471-2105-10-421 PMC2803857 20003500

[34] Saintenac C. , Zhang W., Salcedo A. et al. (2013) Identification of wheat gene Sr35 that confers resistance to Ug99 stem rust race group. *Science*, 341, 783–786. 23811222 10.1126/science.1239022 PMC4748951

[35] Zhu T.T. , Wang L., Rimbert H. et al. (2021) Optical maps refine the bread wheat Triticum aestivum cv. Chinese Spring genome assembly. *Plant J.*, 107, 303–314. 33893684 10.1111/tpj.15289 PMC8360199

[36] Avni R. , Lux T., Minz-Dub A. et al. (2022) Genome sequences of three Aegilops species of the section Sitopsis reveal phylogenetic relationships and provide resources for wheat improvement. *The Plant Journal*, 110, 179–192. 34997796 10.1111/tpj.15664 PMC10138734

[37] Wang L. , Zhu T.T., Rodriguez J.C. et al. (2021) Aegilops tauschii genome assembly Aet v5.0 features greater sequence contiguity and improved annotation. *G3 Genes|Genomes|Genetics*, 11, jkab325. 10.1093/g3journal/jkab325 PMC8664484 34515796

