# Supplementary material for: An online database for einkorn wheat to aid in gene discovery and functional genomics studies
Source: Database (Oxford). 2023 Nov 16;2023:baad079. doi: 10.1093/database/baad079 (PMC10653128; doi:10.1093/database/baad079)
Supplement: baad079_Supp [file baad079_supp.zip › Supplementary_Table1.docx]

Supplementary Table 1. List of Wheat varieties used in this database. A total of 29 varieties were used having hexaploid (16), tetraploid (2) and diploid (11) genomes.

| S.No. | Plant | Ploidy | Reference |
| --- | --- | --- | --- |
| 1. | *Triticum aestivum* cv*.* Mace | Hexaploid | (4) |
| 2. | *T. aestivum* cv*.* LongReach Lancer | Hexaploid | Same as above |
| 3. | *T. aestivum* cv*.* CDC Stanley | Hexaploid | Same as above |
| 4. | *T. aestivum* cv. CDC Landmark | Hexaploid | Same as above |
| 5. | *T. aestivum* cv. Julius | Hexaploid | Same as above |
| 6. | *T. aestivum* cv*.* Norin 61 | Hexaploid | Same as above |
| 7. | *T. aestivum* cv*.* Arina*LrFor* | Hexaploid | Same as above |
| 8. | *T. aestivum* cv*.* PI190962 (spelt wheat) | Hexaploid | Same as above |
| 9. | *T. aestivum* cv*.* Jagger | Hexaploid | Same as above |
| 10. | *T. aestivum* cv. SY Mattis | Hexaploid | Same as above |
| 11. | *T. aestivum* CS-IWGSC ref1 | Hexaploid | (28) |
| 12. | *T. aestivum* CS-IWGSC ref2-1 | Hexaploid | (24) |
| 13. | *T. aestivum* cv*. Fielder* | Hexaploid | (29) |
| 14. | *T. aestivum* cv*. Kariega* | Hexaploid | (30) |
| 15. | *T. aestivum* cv. *Attraktion* | Hexaploid | (31) |
| 16. | *T. aestivum* cv. *Renan* | Hexaploid | (32) |
| 17. | *T. dicoccoides* cv*.* Svevo | Tetraploid | (33) |
| 18. | *T. dicoccoides* cv. *Zavitan2* | Tetraploid | (34) |
| 19. | *Aegilops tauschii* 5.0 | Diploid | (26) |
| 20. | *Ae. tauschii* AY17 | Diploid | (35) |
| 21. | *Ae. tauschii* AY61 | Diploid | Same as above |
| 22. | *Ae. tauschii* T093 | Diploid | Same as above |
| 23. | *Ae. tauschii* XJ02 | Diploid | Same as above |
| 24. | *Ae. tauschii* AL8-78 | Diploid | (36) |
| 25. | *Ae. longissima* | Diploid | (25) |
| 26. | *Ae. speltoides* | Diploid | Same as above |
| 27. | *Ae. sharonensis* | Diploid | (37) |
| 28. | *T. monococcum* TA10622 | Diploid | (13) |
| 29. | *T. monococcum* TA299 | Diploid | Same as above |
