# Supplementary figures and images for: An online database for einkorn wheat to aid in gene discovery and functional genomics studies
Source: Database (Oxford). 2023 Nov 16;2023:baad079. doi: 10.1093/database/baad079 (PMC10653128; doi:10.1093/database/baad079)

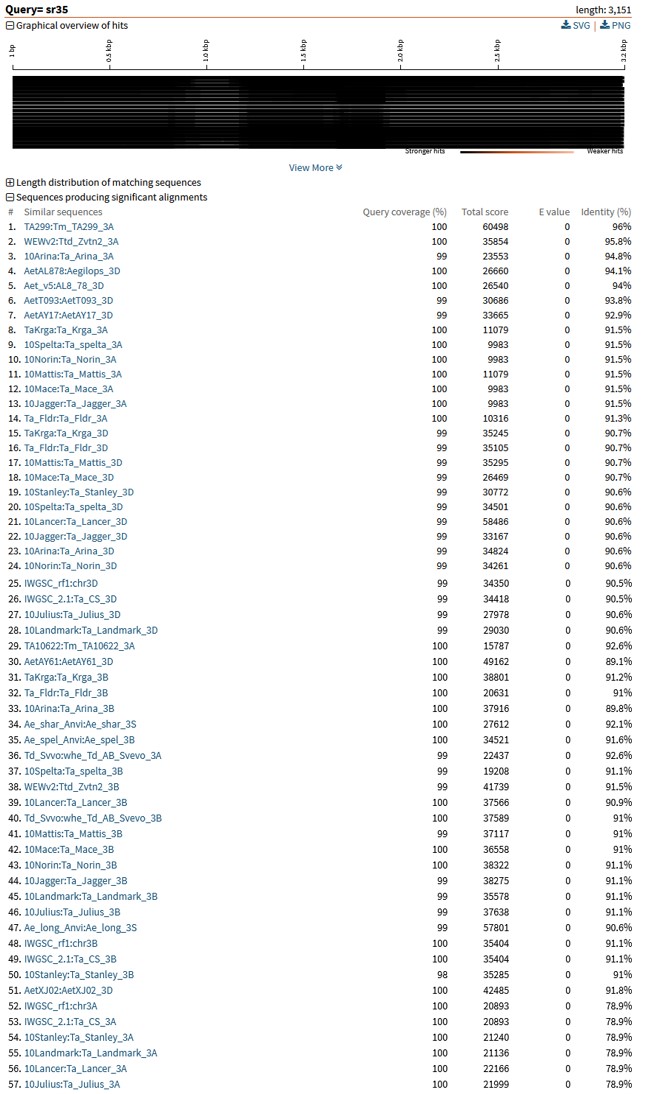

Supplement: baad079_Supp [file baad079_supp.zip › supplementary_figure1.jpg]

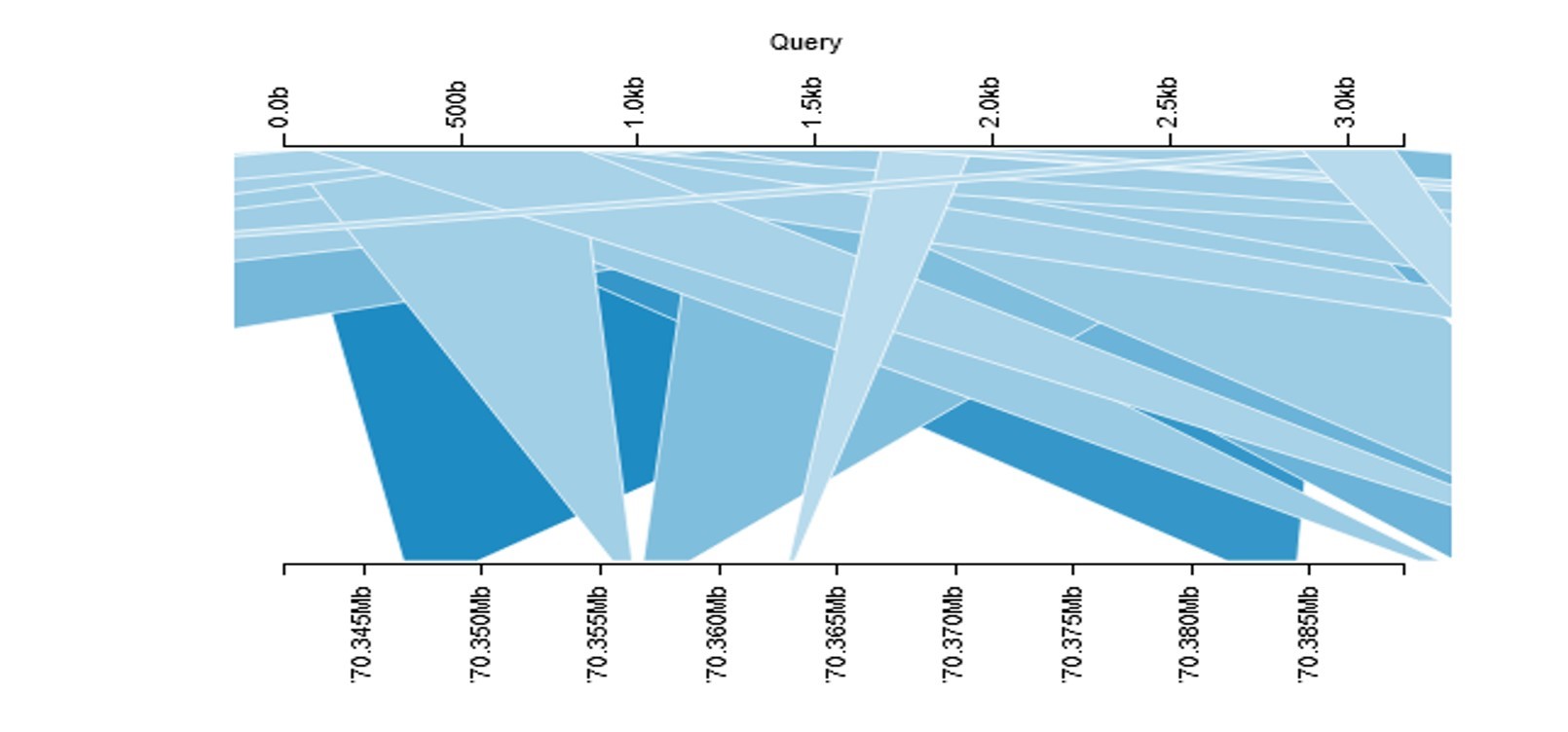

Supplement: baad079_Supp [file baad079_supp.zip › supplementary_figure2.jpg]

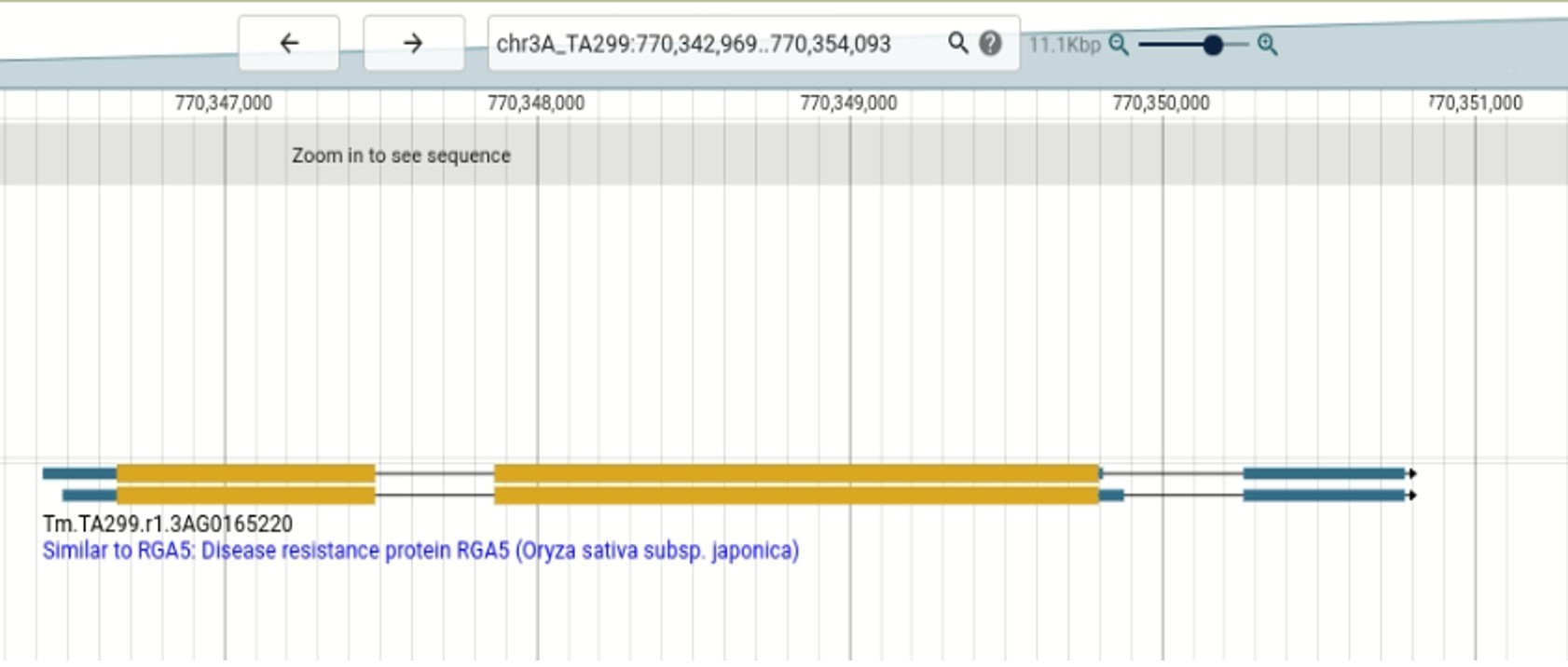

Supplement: baad079_Supp [file baad079_supp.zip › supplementary_figure3.jpg]

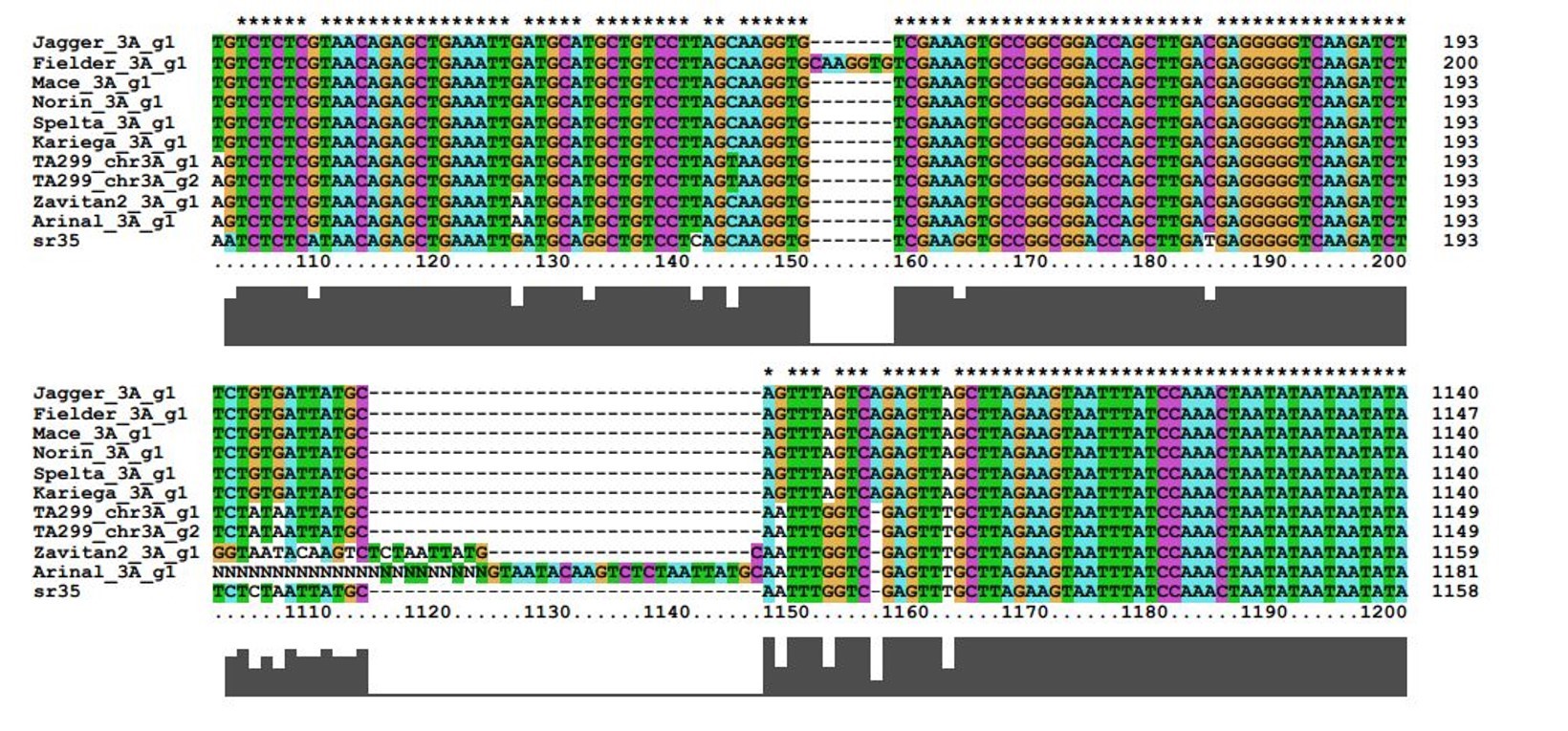

Supplement: baad079_Supp [file baad079_supp.zip › supplementary_figure4.jpg]

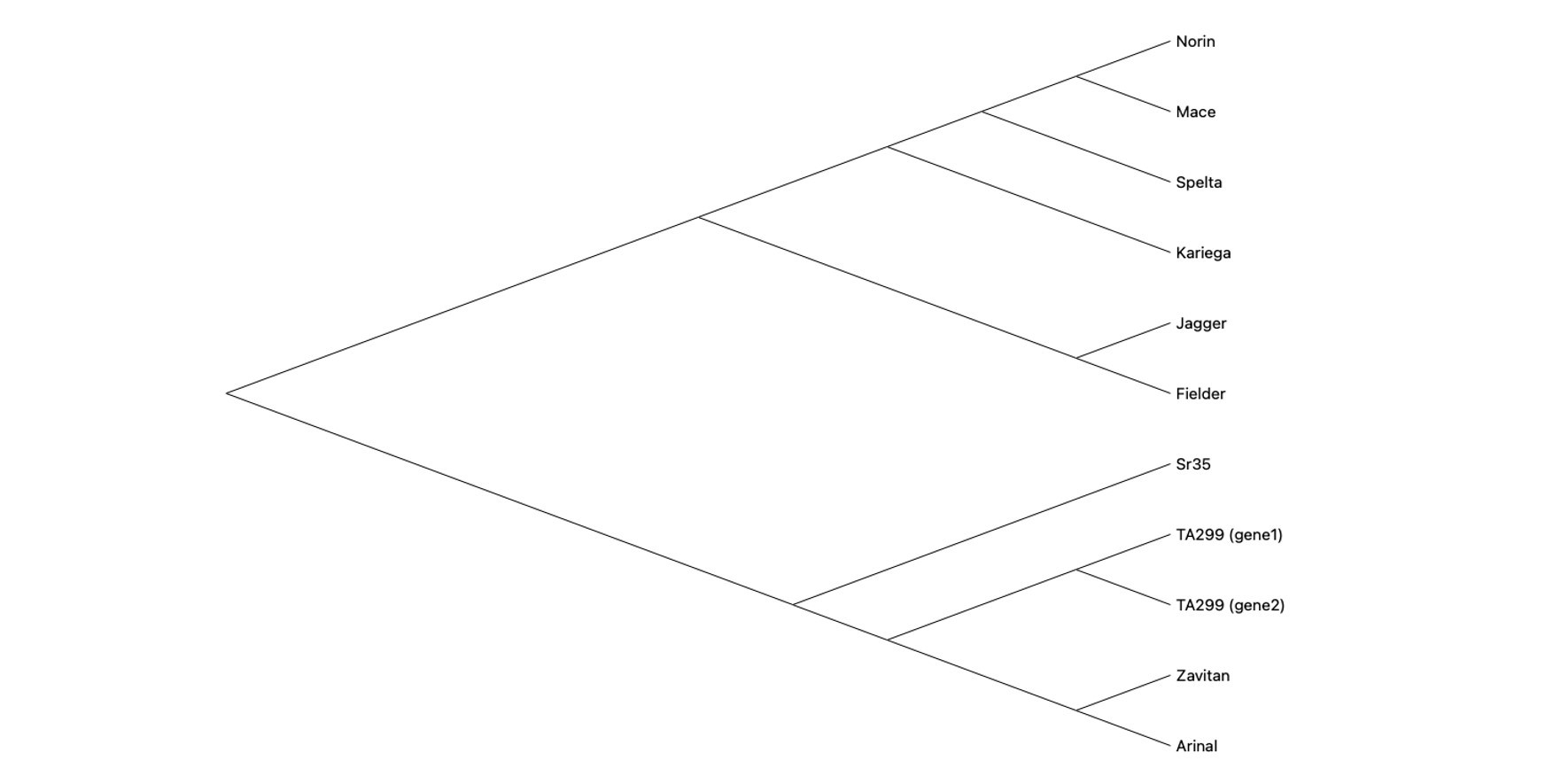

Supplement: baad079_Supp [file baad079_supp.zip › supplementary_figure5.jpg]
